# Characteristics and antimicrobial susceptibility of bacteria causing acute otitis media in children at Vietnam National Children’s Hospital: a cross-sectional study

**DOI:** 10.1093/jacamr/dlaf006

**Published:** 2025-02-14

**Authors:** Hồng Điệp Đỗ, Minh Điển Trần, Tuyết Xương Nguyễn, Thị Bích Thủy Phùng, Thị Bích Ngọc Hoàng, Thị Lan Liên Phạm, Minh Nguyen, Elena DeAngelis, Hữu Tiệp Thân, Khắc Trưởng Nguyễn, Adriana Guzman-Holst

**Affiliations:** Department of Ear - Nose - Throat, Vietnam National Children’s Hospital, Hanoi, Vietnam; Board of Director, Vietnam National Children's Hospital, Hanoi, Vietnam; Department of Ear - Nose - Throat, Vietnam National Children’s Hospital, Hanoi, Vietnam; Department of Molecular Biology for Infectious Diseases, Vietnam National Children's Hospital, Hanoi, Vietnam; Department of Microbiology, Vietnam National Children's Hospital, Hanoi, Vietnam; Training and Research Institute for Child Health, Vietnam National Children's Hospital, Hanoi, Vietnam; GSK, Ho Chi Minh City, Vietnam; GSK, Ho Chi Minh City, Vietnam; Department of Ear - Nose - Throat, Vietnam National Children’s Hospital, Hanoi, Vietnam; Department of Ear - Nose - Throat, Vietnam National Children’s Hospital, Hanoi, Vietnam; GSK, Wavre, Belgium

## Abstract

**Background:**

Acute otitis media (AOM) is a leading cause of healthcare visits, antibiotic prescription and complications in children aged under 5 years. Following the introduction of pneumococcal conjugate vaccines (PCVs), non-typeable *Haemophilus influenzae* (NTHi) has become the most common causative agent for AOM, followed by *Streptococcus pneumoniae* and *Moraxella catarrhalis*. PCVs are not yet included in the National Immunization Program in Vietnam.

**Objectives:**

To determine the frequency and characteristics of the pathogens related to AOM in Vietnam in children ≤5 years old.

**Methods:**

This was a cross-sectional study performed at the Vietnam National Children’s Hospital from October 2021 to December 2023 in children ≤5 years old diagnosed with acute suppurative otitis media. Clinical features of the children were described. Pathogens of interest were identified by culture or real-time PCR (rtPCR). The antibiotic susceptibility profiles of *H. influenzae* and *S. pneumoniae* isolates were determined.

**Results:**

In total, 482 children ≤5 years old were included, of which 70.8% were ≤2 years old and 61% had an history of AOM. The most frequent bacteria isolated were *H. influenzae* (52.1%, 99.6% of which were NTHi) and *S. pneumoniae* (41.1%). Most *S. pneumoniae* isolates were resistant to azithromycin, clarithromycin and cefuroxime. Most *H. influenzae* isolates were resistant to amoxicillin, cefixime, cefuroxime, azithromycin and clarithromycin.

**Conclusions:**

The pathogens most frequently associated with AOM in this study were in line with previous findings. Many isolates were resistant to commonly given oral antibiotics. These results can inform decision-making on AOM prevention and treatment strategies in Vietnam.

## Introduction

Acute otitis media (AOM) is a common bacterial infection in children worldwide. A systematic review on the global burden of AOM estimated its incidence rate was 10.85/100 children-years, 51% occurring in children under 5 years of age.^1^ Previous research concluded that around 80% of children had at least one occurrence of AOM.^2^

AOM involves a complex interaction between microbial pathogens (bacteria and viruses), the structure of the middle ear and nasopharynx, personal immune response and other environmental factors.^3,4^ The most common microbial pathogens causing AOM are *Streptococcus pneumoniae*, *Haemophilus influenzae*, *Moraxella catarrhalis* and *Staphylococcus aureus.*^5–9^  *H. influenzae* has six different capsular serotypes (a to f) and many non-capsular or non-typeable strains, referred to as non-typeable *H. influenzae* (NTHi).^10^ NTHi is more common than other *H. influenzae* strains, causing over 80% of *H. influenzae-*related cases in Asia.^9^

AOM (also referred to as acute suppurative otitis media, ASOM) manifests by the presence of purulent fluid in the middle ear(s) along with the rapid onset of inflammatory signs and symptoms in the middle ear.^11,12^ Common symptoms of AOM include fever, and ear rubbing or ear pain.^12,13^ Though the latter has the highest predictive value for AOM, it is not systematically present.^12,13^ Complications of AOM are caused by the spreading of inflammation to the adjacent areas. They can affect the middle ear (including, but not limited to, impaired hearing, perforation of the tympanic membrane, chronic suppurative otitis media) or the temporal bone (for example, acute mastoiditis, mastoid abscess, facial nerve palsy).^2,14–16^ Intracranial complications encompass meningitis and brain abscess.^2,16^

Antimicrobial resistance of bacterial pathogens is extensively developing, with some instances of multidrug resistance at high doses, resulting in ineffective and/or clinically unsafe treatment. A survey of antibiotic resistance in four countries of south east Asia in 2016–18 concluded that antibiotic resistance varied by country, with the highest rates observed in Vietnam.^17^

With the rising antibiotic resistance rate of pathogens in children with AOM, understanding the characteristics and antibiotic resistance of bacteria regionally is crucial for developing effective treatments and recommendations, setting preventive vaccination strategies, and reducing the risk of antimicrobial resistance.^18^

Our study aimed to describe the frequency and bacteriological characteristics of the pathogens related to AOM in children ≤5 years old at Vietnam National Children’s Hospital (VNCH).

## Materials and methods

### Study design and setting

This was an observational, cross-sectional descriptive study on patients who were examined and treated at the otorhinolaryngology service at VNCH from October 2021 to December 2023. The main objectives were to identify the pathogens of interest by culture or RT–PCR in middle ear fluid from children under 5 years of age with AOM, to describe the clinical features of ASOM, to explore the association of NTHi detection with age and clinical features of patients, and to describe the antibiotic susceptibility profile of pathogens causing AOM.

### Participants and case definition

Children  ≤ 5 years at the time of the study were included if they: (i) had a diagnosis of AOM; (ii) consent to participate was provided by the parents or legal guardians; and (iii) had a successful extraction of middle ear discharge for bacterial culture identification (spontaneous tympanic membrane perforation or during surgery). Patients were excluded if they had AOM without otorrhoea and without surgical indications, if they had taken antibiotics within 72 h with symptom relief, or if they had increased risk factors for AOM, such as congenital or acquired immunodeficiency, cleft palate or craniofacial anomalies. Recurrent AOM was defined as three or more episodes of AOM in 6 months or four or more episodes in 1 year and at least one episode of AOM in the last 6 months, with the bouts of acute infection separated by intervals of full resolution.

### Study procedures

All patients with symptoms suggestive of AOM or diagnosed with ASOM at VNCH were forwarded to the otorhinolaryngology department. Eligibility was assessed by the principal investigator who explained the details of the research to the parents and/or guardians of patients and collected their informed consent. A medical assessment by otoendoscopy was performed by a specialist and all relevant clinical and epidemiological information was recorded in a case-report form (CRF), including data on age, gender, medical history of AOM, medical history of vaccination, clinical symptoms and paraclinical symptoms (Figure S1, available as Supplementary data at *JAC-AMR* Online).

The following clinical characteristics were captured with a predefined and standardized CRF designed for the study: systemic symptoms, signs and symptoms of middle ear, including endoscopic signs.

The purulent discharge from the middle ear was collected. All criteria, research variables and bacterial laboratory results were recorded.

### Bacterial testing methods and techniques

Bacterial identification was performed by two methods: culture and rtPCR. *H. influenzae* capsular serotypes were determined by multiplex rtPCR. The antibiotic susceptibility profiles of *H. influenzae* and *S. pneumoniae* isolates were established based on the antibiotic MICs on culture plates.

#### Middle ear fluid extraction

Sterile swabs were used to extract the purulent discharge near the tympanic membrane to collect two different specimens. Samples were sent to the microbiology department and the molecular biology department within 30 min of extraction.

#### Culture identification

Plates with chocolate agar and blood agar were inoculated with the swab containing the specimen. If the culture was positive within 3 days of culture, the identification and susceptibility testing was performed using the VITEK 2 system.

#### Antibiotic susceptibility

After successful bacterial identification, the antibiotic susceptibility testing protocol was applied. For *H. influenzae* and *S. pneumoniae*, the MIC determination protocol was applied for amoxicillin, amoxicillin/clavulanate, cefuroxime, ceftriaxone, azithromycin, clarithromycin and levofloxacin. For *H. influenzae,* cefixime was also evaluated. After 18–24 h, the MIC values were evaluated based on CLSI standards^19^ for antibiotics (and EUCAST^20^ for amoxicillin in *H. influenzae*).

#### RT–PCR for *H. influenzae, S. pneumoniae and M. catarrhalis*

Specimens with negative culture identification results were tested by rtPCR. DNA was extracted with the MagNA Pure 96 DNA and Viral NA Small Volume Kit, using the MagNA Pure 96 System (Roche, Indianapolis, IN, USA). rtPCR was performed with the Applied Biosystems 7500 Fast Real-Time PCR System (Thermo Fisher Scientific, Waltham, MA, USA). Primers and probes were synthesized by the IDT company (Integrated DNA Technologies, Coralville, IA, USA), targeting the genes for autolysin (*lytA*), L-fuculokinase (*fucK*) and outer membrane protein (*copB*), specific to *S. pneumoniae*, *H. influenzae* and *M. catarrhalis*, respectively.^21^

All samples positive for *H. influenzae* were tested for NTHi using rtPCR. Direct rtPCR primers and probes for *H. influenzae* species and serotype assays followed methods described by Marasini *et al*.^22^

#### Molecular testing procedure for NTHi detection

Culture-negative specimens were subjected to monoprimer rtPCR targeting the *phoB* gene specific to *H. influenzae* species. Next, to determine capsular serotypes (a–f) of *H. influenzae*, *H. influenzae* rtPCR-positive specimens were further tested with multiplex rtPCR targeting serotype-specific genes in region II of the capsule biosynthesis locus. Isolates that were *H. influenzae* positive but negative to all serotypes (a–f) by multiplex rtPCR were considered NTHi. Primers/probes and rtPCR programs were described in the study of Marasini *et al*.^22^

#### Positive-control preparation procedure

Positive controls were *H. influenzae* strain ATCC 9006 (type a), ATCC 9795 (type b), ATCC 9007 (type c), ATCC 9332 (type d), ATCC 8142 (type e) and ATCC 9833 (type f). Positive-control preparation for rtPCR was processed from freeze-dried strains following the manufacturer’s instructions. Briefly, the rehydrated pellet of *H. influenzae* strains was aseptically transferred into #814 broth, then grown at 37°C for 24 h. The primary broth was then transferred into an #814 agar plate and grown at 37°C for 24–48 h. A suspension of *H. influenzae* strain from single colonies was prepared using the 0.5 McFarland standard to extract their genome.

### Statistical methods

#### Study size

The sample size was calculated as follows^23^:


$$n=Z_{\left(1-\frac{\alpha }{2}\right)}^{2}\frac{\;p(1-p)}{d^{2}}$$

where *n* is the minimal sample size, α is the statistical significance level (α = 0.05, corresponding to 95% confidence level), Z is the Z-score for a 95% confidence level, found to be 1.96, d is the absolute precision, with a chosen value of 0.02, and p is the estimate of the distribution rate of common bacteria causing AOM in children based on previous studies: 22.2% for *H. influenzae*, 26.4% for *S. pneumoniae* and 5.4% for *M. catarrhalis*.^9^ The sample size was calculated for each bacterial agent, and the largest sample size calculated among the above bacteria was selected. Using *P* = 0.05, the minimum sample size was 457.

#### Results analysis

A descriptive analysis was performed to assess the relative frequency of NTHi detection and the other pathogens overall and stratified by age (under or above 2 years of age) in the study population. The antibiotic susceptibility was described by pathogen in the overall population.

The chi-squared test was used to measure the strength of a relationship between NTHi with other variables as age and clinical AOM features; *P* values below 0.05 were considered statistically significant.

## Results

### Demographics and medical history

A total of 482 AOM patients meeting all selection criteria were recruited for this study. The average age of children at AOM onset was 19.4 months; the youngest patient was 1 month old. Children between 0 and 24 months of age accounted for 70.8% of all study patients, and 51.2% of patients were male (Table 1; Figure S2).

**Table 1.** Demographic characteristics and medical history of study participants

Characteristic	*n*	%
Age (months)		
0–12	126	26.2
>12–24	215	44.6
>24–36	110	22.8
>36–48	28	5.8
>48–60	3	0.6
Gender		
Male	247	51.2
Female	235	48.8
History of AOM		
No	188	39.0
Yes	294	61.0
Recurrent AOM	95	32.3
Surgery related to otitis media	61	20.7
Not specified	128	43.5
History of pneumococcal conjugate vaccination		
No	217	45.0
Yes	238	49.4
Unknown	27	5.6
Total	482	100

AOM, acute otitis media; *n*, number of participants in the category.

In total, 294 (61%) research patients had previously had at least one episode of AOM. Of these patients, recurrent AOM accounted for 95 cases (32.3%); meanwhile, 61 cases (20.7%) had a history of medical interventions related to AOM, such as myringotomy and tympanostomy tube placement. Half of the research patients had previously received pneumococcal conjugate vaccination.

### Clinical characteristics of AOM and NTHi

Most children had AOM in both ears (Table 2). The rate of unilateral and bilateral AOM differed significantly between age groups. The most common symptoms of AOM included fever, ear pain and ear rubbing (Table 2). Amongst the patients, 466 had spontaneous purulent discharge, 16 (3.3%) had AOM with complications requiring surgery, including one case with facial paresis undergoing tympanostomy tube placement, and 15 cases with mastoid abscess undergoing mastoidectomy and tympanostomy tube placement.

**Table 2.** Clinical signs and symptoms and diagnostic characteristics of AOM in study participants

Signs	All (*N* = 482)	0–2 years (*n* = 341)	>2 years (*n* = 141)	*P* value
Clinical signs and symptoms				
Unilateral AOM	115	69	46	<0.05
Bilateral AOM	367	272	95	
Otalgia (ear pain)	224	128	96	
Pulling or rubbing at the ears	196	149	47	
Fever				
No fever	212	155	57	
Fever, <39°C	135	95	40	
Fever, ≥39°C	135	91	44	
Diagnostic characteristics of AOM				
AOM with spontaneous tympanic membrane perforation	466	329	137	0.7
AOM-related facial nerve palsy or mastoid abscess	16	12	4	
AOM-related facial nerve palsy	1	1	0	
AOM-related mastoid abscess	15	11	4	

AOM, acute otitis media; *N*, Total number of participants in each age group and overall; *n*, number of participants in the category.

The rate of NTHi was significantly higher in the recurrent AOM group than in the non-recurrent AOM group (*P* = 0.007, Table S1). Fever was significantly more frequent in the NTHi group, compared with other pathogens (*P* = 0.02).

### AOM aetiology

**Figure 1.**
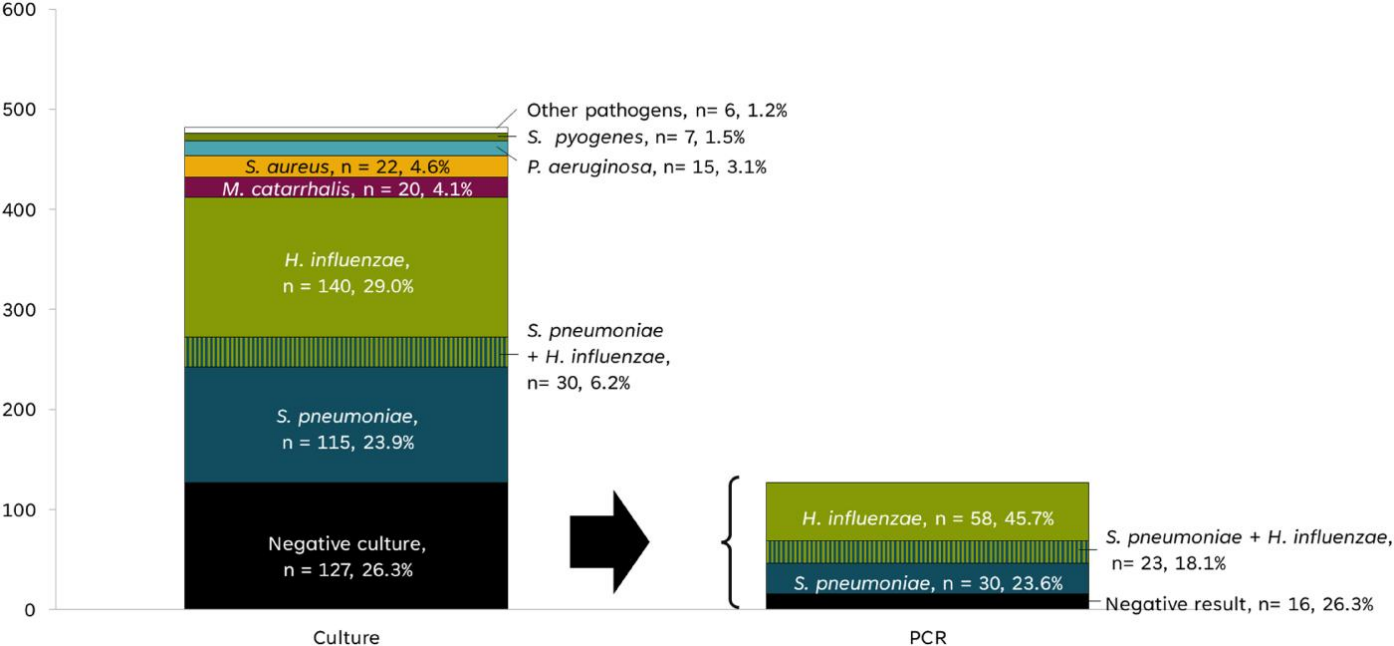
Aetiology of AOM identified by culture or PCR. AOM, acute otitis media; n, number of samples in the culture; PCR, polymerase chain reaction.

Bacterial isolation by culture permitted the identification of 385 (79.9%) positive isolations out of 482 samples, with 30 samples yielding positive cultures for two different bacterial species. Among the successfully isolated pathogens, *H. influenzae* and *S. pneumoniae* accounted for two-thirds of the cultured samples (35.3% and 30.1%, respectively) (Table S2). *S. aureus*, *M. catarrhalis*, *Pseudomonas aeruginosa*, and *Streptococcus pyogenes* each represented less than 5% of the total identified isolates. For the 127 specimens with a negative bacterial culture result, rtPCR was conducted to detect *S. pneumoniae*, *H. influenzae* and *M. catarrhalis*. *H. influenzae* was observed in 58 samples, and *S. pneumoniae* in 30 samples; 23 samples were positive for both pathogens (Figure 1).

*H. influenzae* isolates identified by culture or rtPCR (a total of 251 samples) were subjected to capsular serotyping. Out of 251 isolates, 250 (99.6%) were NTHi, and 1 (0.4%) was *H. influenzae* type b (Table S2).

*S. pneumoniae* was isolated in most AOM cases with complications (Table S3), whereas *H. influenzae* (including both typeable *H. influenzae* and NTHi) was most frequently identified in recurrent AOM cases (Table S4).

### Antibiotic susceptibility

**Figure 2.**
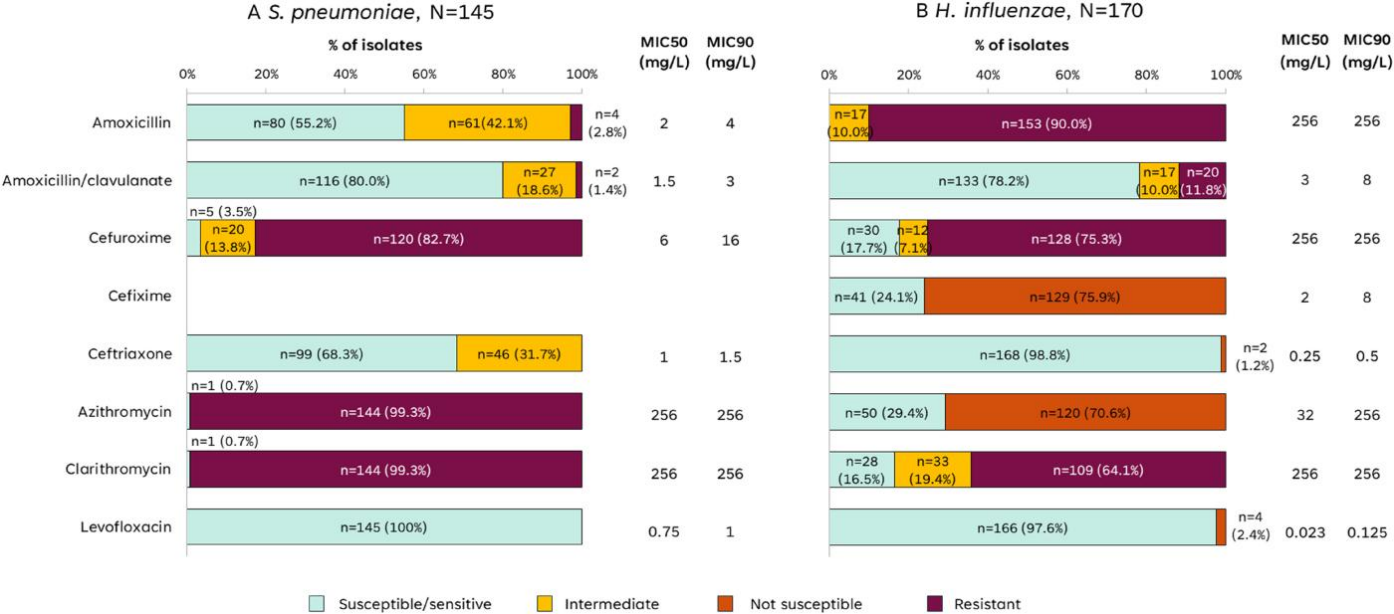
Antibiotic susceptibility of *S. pneumoniae* and *H. influenzae* isolates. MIC, minimum inhibitory concentration; n, number of isolates in the category; N, total number of isolates of the species.

Antibiotic susceptibility was tested in 145 isolates of *S. pneumoniae*. All *S. pneumoniae* isolates were susceptible to levofloxacin (Figure 2). *S. pneumoniae* were mostly susceptible to amoxicillin/clavulanate (80.0%), ceftriaxone (68.3%) and amoxicillin (55.2%). Almost all *S. pneumoniae* isolates were resistant to azithromycin, clarithromycin and cefuroxime.

Antibiotic susceptibility was tested in 170 isolates of *H. influenzae*. Susceptibility to ceftriaxone and levofloxacin was found in almost all *H. influenzae* isolates; 78.2% were susceptible to amoxicillin/clavulanate. *H. influenzae* isolates were not susceptible to amoxicillin alone, and their susceptibility to other tested antibiotics was below 30%.

## Discussion

Our study describes AOM causative agents during 2021–23 in VNCH. The most commonly isolated bacteria in middle ear fluid samples in patients presenting with AOM were NTHi and *S. pneumoniae*, combining both culture and RT–PCR methods. NTHi predominated in cases of recurrent AOM, while *S. pneumoniae* was predominant in complicated AOM cases. Most AOM cases occurred in children aged 2 years and younger (70.8%), in line with previous studies across the world.^24–26^ Young children are known to be more prone to AOM than older children and adults, due to the immaturity of both their immune system and Eustachian tube structure, and frequent exposure to pathogens.^27–31^

In recent years, there has been a balance in the prevalence of *S. pneumoniae* and *H. influenzae* as causes of AOM in children, due to the introduction of the pneumococcal conjugate vaccine.^9,32–34^ In our study, *S. pneumoniae* ranked close second, but was the main pathogen causing complicated AOM (15 out of 16 cases), which was consistent with previous reports.^35^ Other bacterial isolates in our research were *S. aureus*, *M. catarrhalis*, *P. aeruginosa* and *S. pyogenes*. *M. catarrhalis* is commonly found in the nasopharynx of children, with a significantly lower risk of complications such as tympanic membrane perforation and acute mastoiditis, compared with *H. influenzae* and *S. pneumoniae*.^36^ In our study, *M. catarrhalis* was observed in one case with acute mastoiditis, and one case with recurrent AOM. *S. aureus* is an important upper respiratory tract pathogen originating from the nasopharynx.^37^  *S. aureus* and *P. aeruginosa* are saprophytic bacteria found in the external ear canal; their presence may either be regarded as a contamination during discharge extraction, or as a simultaneous infection.^38^

Our study utilized the PCR method, which enhanced the detection rate of bacteria, particularly in identifying NTHi. NTHi was the most common pathogen for AOM in children under 5 years in VNCH, with 250 NTHi isolates and only one instance of *H. influenzae* type b out of 251 *H. influenzae*-positive samples. As *H. influenzae* type b vaccination was implemented in Vietnam in 2010 as part of the pentavalent vaccination,^39^ the number of cases caused by this pathogen has significantly decreased. These results complement recent studies in Asia, which concluded that NTHi predominates (over 80%) in patients with *H. influenzae*-related AOM.^9^

In our study, *S. pneumoniae* isolates were almost entirely resistant to azithromycin and clarithromycin, and highly resistant to cefuroxime. *S. pneumoniae* was susceptible to levofloxacin, ceftriaxone and amoxicillin, and highly susceptible to amoxicillin/clavulanate. A survey by Torumkuney *et al*.^17^ on antibiotic resistance status from 2016 to 2018 reported that the level of antibiotic resistance in *S. pneumoniae* isolates in community-acquired infections in Vietnam was the highest among the four studied southeast Asian countries, with a susceptibility rate of 60% for amoxicillin, amoxicillin/clavulanate and ceftriaxone, but less than 14% for most other antibiotics. A study conducted in Taiwan from 2010 to 2015 by Cho *et al*.^40^ reported only 30.8% and 32.0% of *S. pneumoniae* being susceptible to amoxicillin and penicillin, respectively, and only 62.8% and 68.0% of pneumococcal strains susceptible to cefotaxime and ceftriaxone, respectively.

*H. influenzae* isolates were no longer susceptible to amoxicillin, and less susceptible to azithromycin, cefixime, cefuroxime and clarithromycin in this analysis. Almost all *H. influenzae* isolates were susceptible to ceftriaxone and levofloxacin, but showed a high susceptibility rate to amoxicillin/clavulanate. There is a difference between our results and other studies regarding the antibiotic susceptibility of *H. influenzae*. Torumkuney *et al*.^17^ reported *H. influenzae* isolates from community-acquired infections having a susceptibility rate of over 85% to amoxicillin/clavulanate (95.5%), ceftriaxone (100%) and macrolides (87.6%–89.9%). The susceptibility rates were 57.3%–59.6% for fluoroquinolones, and 70.8% for cefixime. Cho *et al*.^40^ reported a high level of resistance to ampicillin in NTHi strains; however, they were susceptible to cefuroxime (77%) and amoxicillin/clavulanate (82.0%), and highly susceptible to cefotaxime and ceftriaxone (98.4%). Hence, the antibiotic resistance characteristics change over time and vary between regions. NTHi also emerged as the predominant causative agent in recurrent AOM in our study. The antibiotic resistance of NTHi contributes to recurrent AOM and treatment failure in children, posing significant challenges for clinicians and becoming an increasingly alarming public health issue.^41^

Limitations to this study include those common to cross-sectional studies. It provides an overview of aetiological agents involved in AOM in children aged 0–5 years presenting at VNCH during 2021–23 but does not capture the possible evolution of the proportion of the principal causative agents of AOM in the population or their antibiotic resistance profile over time. We did not serotype *S. pneumoniae* isolates. It is not possible to exclude the fact that some AOM cases may have been caused by pathogens not tested for, or not detected by our protocol. Further, these results may not be generalizable to settings outside Vietnam as the epidemiology of AOM is influenced by geography.^18^

### Conclusions

NTHi and *S. pneumoniae* are the two most common bacteria causing AOM in children in Vietnam. Amoxicillin/clavulanate is recommended as the first-line treatment for AOM due to its high efficacy against amoxicillin-resistant bacterial strains. Furthermore, this study highlights the importance of developing and implementing preventive strategies for AOM, including the use of pneumococcal and NTHi vaccines. The expanded immunization programme is expected to introduce the pneumococcal conjugate vaccine in 2025, offering hope for a significant reduction in disease incidence. Regular updates on the antibiotic resistance profiles of AOM-causing pathogens are essential to support clinical recommendations for treatment and prevention.

## Supplementary Material

dlaf006_Supplementary_Data

## Data Availability

Please refer to GSK weblink to access GSK’s data-sharing policies and, as applicable, seek anonymised subject-level data via the link https://www.gsk-studyregister.com/en/.

## Supplementary data

Figures S1 and S2 and Tables S1 to S4 are available as Supplementary data at *JAC-AMR* Online.
