# Supplementary material for: Characteristics and antimicrobial susceptibility of bacteria causing acute otitis media in children at Vietnam National Children’s Hospital: a cross-sectional study
Source: JAC Antimicrob Resist. 2025 Feb 14;7(1):dlaf006. doi: 10.1093/jacamr/dlaf006 (PMC11826232; doi:10.1093/jacamr/dlaf006)
Supplement: dlaf006_Supplementary_Data [file dlaf006_supplementary_data.docx]

# Supplementary material

**Table S1. Clinical characteristics of NTHi positive patients & association of**

**NTHi detection with age and clinical features (n=250)**

| **Characteristics** | **Subcategories** | **All** | **NTHi (n,%)** | **Others (n,%)** | **p-value** |
| --- | --- | --- | --- | --- | --- |
| Age | 0-2 years | 341 | 183 (53.7) | 158 (46.3) | p = 0.2 |
|  | >2 years | 141 | 67 (47.5) | 74 (52.5) |  |
| Recurrent AOM | Yes | 95 | 61 (64.2) | 34 (35.8) | p = 0.007 |
|  | No | 387 | 189 (48.8) | 198 (51.2) |  |
| Diagnosis | AOM with spontaneous TM perforation | 466 | 248 (53.2) | 218 (46.8) | p = 0.001 |
|  | AOM-related facial nerve palsy or mastoid abscess | 16 | 2 (12.5) | 14 (87.5) |  |
| AOM | Unilateral AOM | 115 | 53 (46.1) | 62 (53.9) | p = 0.1 |
|  | Bilateral AOM | 367 | 197 (53.7) | 170 (46.3) |  |
| Fever | Yes | 212 | 127 (60.0) | 85 (40.0) | p = 0.02 |
|  | No | 270 | 123 (45.6) | 147 (54.4) |  |
| **N** |  | **482** | **250** | **232** |  |

AOM: acute otitis media, N: number of samples, n: number of samples in the category, NTHi: non-typeable *H. influenzae*, TM: tympanic membrane**Table S2. Etiology of AOM in children 0-5 years old**

| **Method, bacteria** | **n** | **%^a^** |
| --- | --- | --- |
| **Bacteria isolated by culture method (N = 482)** | | |
| *S. pneumoniae* | 145 | 30.1 |
| *H. influenzae* | 170 | 35.3 |
| *M. catarrhalis* | 20 | 4.1 |
| *S. aureus* | 22 | 4.6 |
| *P. aeruginosa* | 15 | 3.1 |
| *S. pyogenes* | 7 | 1.5 |
| Other pathogens | 6 | 1.2 |
| Negative culture | 127 | 26.3 |
| **Bacterial identification result by real-time PCR (N=127)** | | |
| *S. pneumoniae* | 53 | 41.7 |
| *H. influenzae* | 81 | 63.8 |
| *M. catarrhalis* | 0 | 0 |
| Negative result | 16 | 12.6 |
| ***H. influenzae* strain identification (N= 251)** | | |
| NTHi | 250 | 99.6 |
| *H. influenzae type b* | 1 | 0.4 |

^a^ Denominator: Total N

AOM: acute otitis media, N: Number of samples, n: Number of samples in the category, NTHi: non-typeable *H. influenzae*, PCR: polymerase chain reaction

**Table S3. Bacterial identification in cases of AOM with complications**

| **Method** | **Results** | **AOM-related mastoid abscess (n)** | **AOM-related facial nerve palsy (n)** |
| --- | --- | --- | --- |
| Bacterial culture | *S. pneumoniae* | 8 | 1 |
|  | *H. influenzae* | 0 | 0 |
|  | *M. catarrhalis* | 1 | 0 |
|  | Negative | 6 | 0 |
| Real-time PCR  (for samples with negative culture) | *S. pneumoniae* | 6 | 0 |
|  | *H. influenzae* | 2 | 0 |
|  | *M. catarrhalis* | 0 | 0 |

AOM: acute otitis media, n: number of isolates in each category,

PCR: polymerase chain reaction

**Table S4. Bacterial identification in cases of recurrent AOM**

|  | **n, %^a^** |
| --- | --- |
| **Bacteria culture** |  |
| *H. influenzae* | 28 (29.5%) |
| *S. pneumoniae* | 15 (15.8%) |
| *P. aeruginosa* | 5 (5.3%) |
| *S. aureus* | 4 (4.2%) |
| *S. pyogenes* | 1 (1.1%) |
| *H. influenzae* + *S. pneumoniae* | 1 (1.1%) |
| *H. influenzae +M. catarrhalis* | 1 (1.1%) |
| Other pathogens | 1 (1.1%) |
| Negative culture | 39 (41.1%) |
| **Real-time PCR *(For cases with negative culture)*** |  |
| NTHi | 21 (53.8%) |
| *S. pneumoniae* | 5 (12.8%) |
| NTHi*+ S. pneumoniae* | 10 (25.6%) |
| *M. catarrhalis* | 0 (0.0%) |
| Negative | 3 (7.7%) |

^a^ For bacterial culture, the percentages are calculated with the total number of recurrent AOM cases (N=95) as denominator. For PCR, the percentages are calculated based on the number of recurrent AOM cases with negative bacterial culture (N=39) as denominator.

AOM: acute otitis media, PCR: polymerase chain reaction, n: number of isolates in each category, NTHi: non-typeable *H. influenz*

**
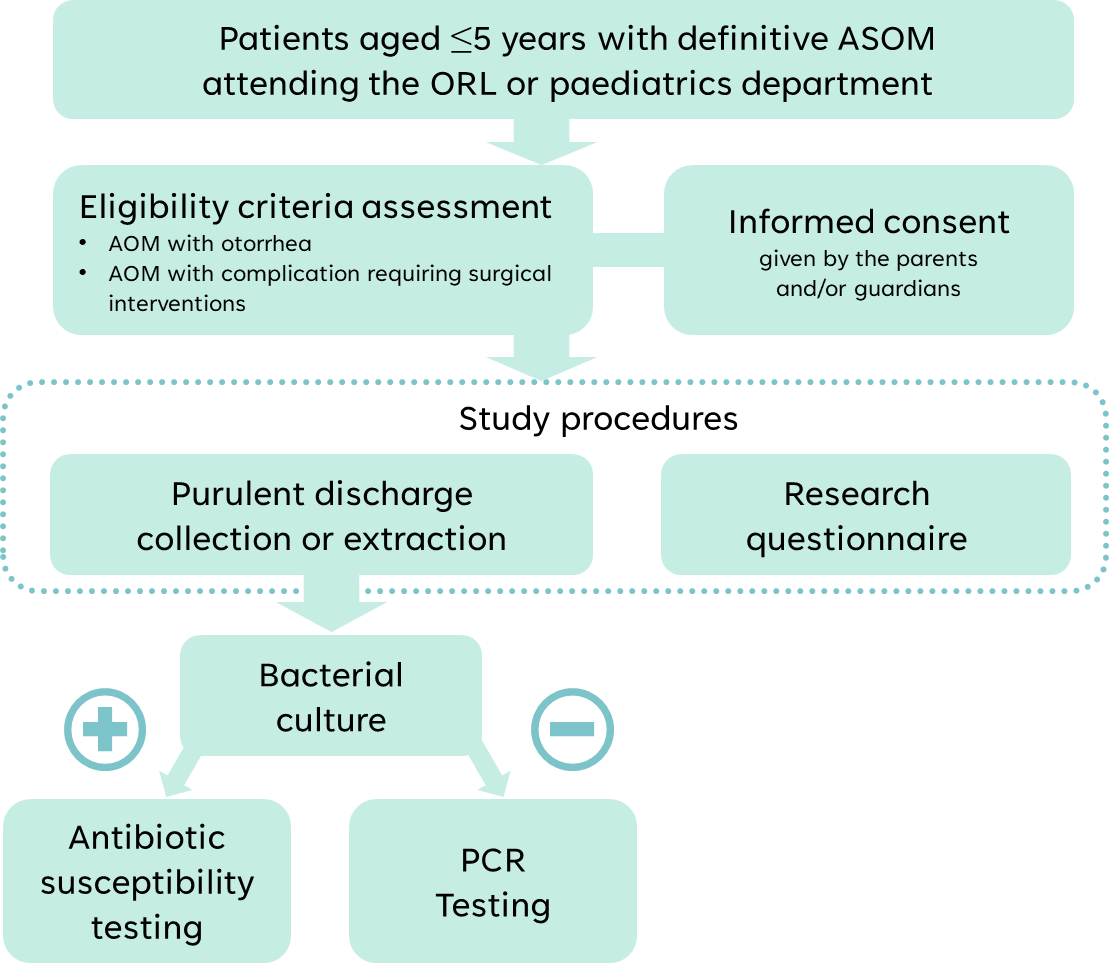
**

**Figure S1. Study flow.** ASOM: acute suppurative otitis media, ORL: otorhinolaryngology;

AOM: acute otitis media, PCR: polymerase chain reaction

**
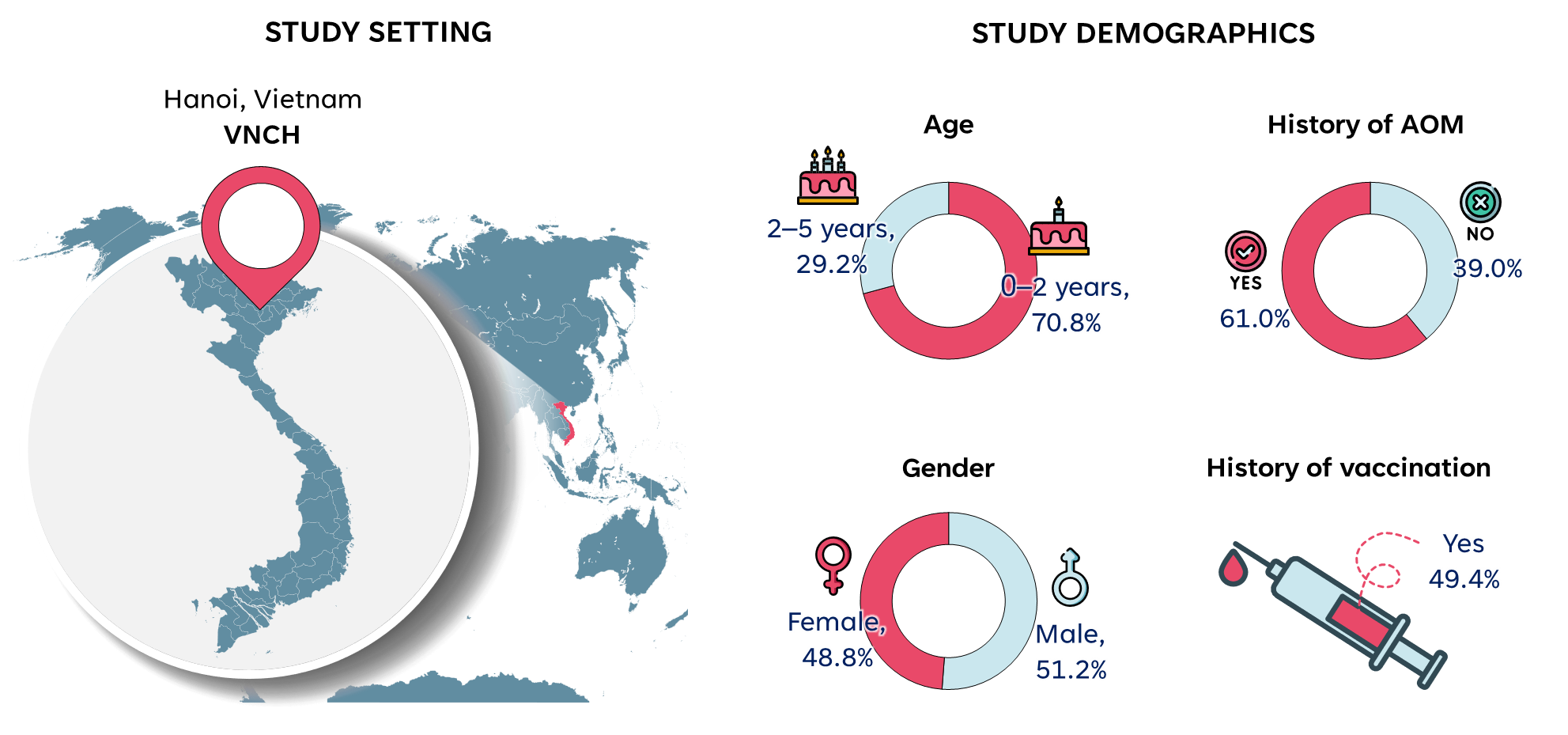
**

**Figure S2. Study settings and demographics of participants.** AOM: acute otitis media, VNCH: Vietnam National Children’s Hospital
